# Hypofractionated radiotherapy versus conventional radiotherapy in patients with intermediate- to high-risk localized prostate cancer: a meta-analysis of randomized controlled trials

**DOI:** 10.1186/s12885-019-6285-x

**Published:** 2019-11-08

**Authors:** Wei Guo, Yun-Chuan Sun, Jian-Qiang Bi, Xin-Ying He, Li Xiao

**Affiliations:** Department of Radiation Oncology, Hebei Province Cangzhou Hospital of Integrated Traditional and Western Medicine, Cangzhou, 061000 Hebei China

**Keywords:** Prostate cancer, Hypofractionated radiotherapy, Conventional radiotherapy, Efficacy, Adverse event

## Abstract

**Background:**

Prostate cancer is one of the most common cancers in the world. The results of treatment after hypofractionated radiotherapy only have been reported from several small randomized clinical trials. Therefore, we conducted a meta-analysis to compare clinical outcomes of hypofractionated radiotherapy versus conventional radiotherapy in the treatment of intermediate- to high-risk localized prostate cancer.

**Methods:**

Relevant studies were identified through searching related databases till August 2018. Hazard ratio (HR) or risk ratio (RR) with its corresponding 95% confidence interval (CI) was used as pooled statistics for all analyses.

**Results:**

The meta-analysis results showed that overall survival (HR = 1.12, 95% CI: 0.93–1.35, *p* = 0.219) and prostate cancer-specific survival (HR = 1.29, 95% CI: 0.42–3.95, *p* = 0.661) were similar in two groups. The pooled data showed that biochemical failure was RR = 0.90, 95% CI: 0.76–1.07, *p* = 0.248. The incidence of acute adverse gastrointestinal events (grade ≥ 2) was higher in the hypofractionated radiotherapy (RR = 1.70, 95% CI: 1.12–2.56, *p* = 0.012); conversely, for late grade ≥ 2 gastrointestinal adverse events, a significant increase in the conventional radiotherapy was found (RR = 0.75, 95% CI: 0.61–0.91, *p* = 0.003). Acute (RR = 1.01, 95% CI: 0.89–1.15, *p* = 0.894) and late (RR = 0.98, 95% CI: 0.86–1.10, *p* = 0.692) genitourinary adverse events (grade ≥ 2) were similar for both treatment groups.

**Conclusion:**

Results suggest that the efficacy and risk for adverse events are comparable for hypofractionated radiotherapy and conventional radiotherapy in the treatment of intermediate- to high-risk localized prostate cancer.

## Background

Prostate cancer (PCa) is one of the most common cancers in the world, especially in North America and Western Europe [1], with over 50% of patients suffering from intermediate- to high-risk localized PCa [2, 3]. On the basis of the results of previous studies, external-beam radiation therapy (EBRT) combined with androgen deprivation therapy (ADT) is a standard treatment for patients with intermediate- to high-risk PCa [4, 5]. Compared with a dose of 75.6 to 79.2Gy for low-risk patients, doses up to 81Gy in form of conventional fractionation schedules have been recommended for patients with intermediate- to high-risk PCa [6–8]. However, conventionally fractionated dose escalation protracts treatment time, which could possibly increase side effects and yield lower treatment efficacy.

In ideal conditions, radiotherapy dose fractionation schedules should take into account the sensitivity to radiation of the tumor relative to nearby non-tumor tissues. Accumulating evidence shows that the α/β ratio for PCa is low and range from 0.9 to 2.2 Gy [9]. Radiobiological theory suggests that hypofractionated radiation schedules applied in fewer fractions and with larger single doses could increase treatment effects [10]. Further, hypofractionated radiotherapy with single dose≥2.5 Gy per fraction could theoretically maintain high biologically effective doses, while not increasing acute and late adverse events, but efficiently shortening the treatment time. Such outcome would translate into higher treatment capacity and could potentially reduce treatment cost [11].

The results of treatment after hypofractionated radiotherapy have only been reported from several small randomized trials [12, 13]. The efficacy and adverse events of hypofractionated radiotherapy seemed to be comparable with conventional schedules in the treatment of intermediate- to high-risk PCa. However, small sample size trials might have biased results, although no significant effect of publication bias was detected. Lastly, we pooled the relevant outcomes of randomized trials and compared the efficacy and adverse events profile of hypofractionated with those of conventional radiotherapy for intermediate- to high-risk localized PCa.

## Methods

### Literature search

This meta-analysis was conducted according to Preferred Reporting Items For Systematic Reviews and Meta-analyses guidelines (PRISMA) [14]. As this meta-analysis was performed based on the published data, ethics committee and/or institutional board approval was not required. Our literature search was performed via Pubmed, Embase and Web of Science databases. The last search was updated to August 2018. The search strategy was: “prostatic neoplasms” (MeSH Terms), “radiotherapy” (MeSH Terms), and “hypofractionated” (All Fields). At the same time, we also checked abstracts published in major academic conferences. The references of studies included were screened to locate potentially eligible articles.

### Study selection

The selected studies should meet the following eligibility criteria: (1) comparison of the use of hypofractionated (ie, dose per fraction range from 2.4–4.0 Gy) with that of conventional radiotherapy (1.8–2.0 Gy per fraction) for intermediate- to high-risk PCa; (2) clear description of applied case selection criteria; (3) reported data allows calculating hazard ratio (HR) or risk ratio (RR) with its corresponding 95% confidence interval (CI) or alternatively these could be computed according to Tierney’s method [15]; (4) published as full-text articles; (5) published in English language. The exclusion criteria were: (1) patients have received previous pelvic radiotherapy or radical prostatectomy; (2) animal studies; (4) letters, conference abstracts or review articles.

### Data extraction

Two investigators (W.G. And L.X.) independently extracted the following data from the eligible studies using a predefined protocol: name of the first author, country, sample size, radiotherapy methods, radiotherapy schedule, androgen deprivation therapy (ADT) and clinical outcome measures. Discrepancies between the two reviewers were settled by the third investigator (Y.C.S. and X.Y.H.).

### Statistical analysis

HRs and RRs with 95% CIs for clinical outcome measures were directly obtained from each study if available or were calculated from raw data using the method reported by Tierney et al. [15]. The Cochran’s Q test and Higgins I-squared statistic were used to evaluate the heterogeneity of pooled results. If I^2^ >50% and P for heterogeneity < 0.1, which show significant heterogeneity, the random-effect model was used; otherwise, the fixed-effects model was conducted. Sensitivity analyses were performed to evaluate the impact of individual studies on the overall estimate. Begg’s funnel plot was assessed to find publication bias. All data were analyzed through the STATA 12.0 software (Stata Corp, College Station, TX, USA). A *p*-value < 0.05 was considered as statistically significant.

## Results

### Study characteristics

**Fig. 1:**
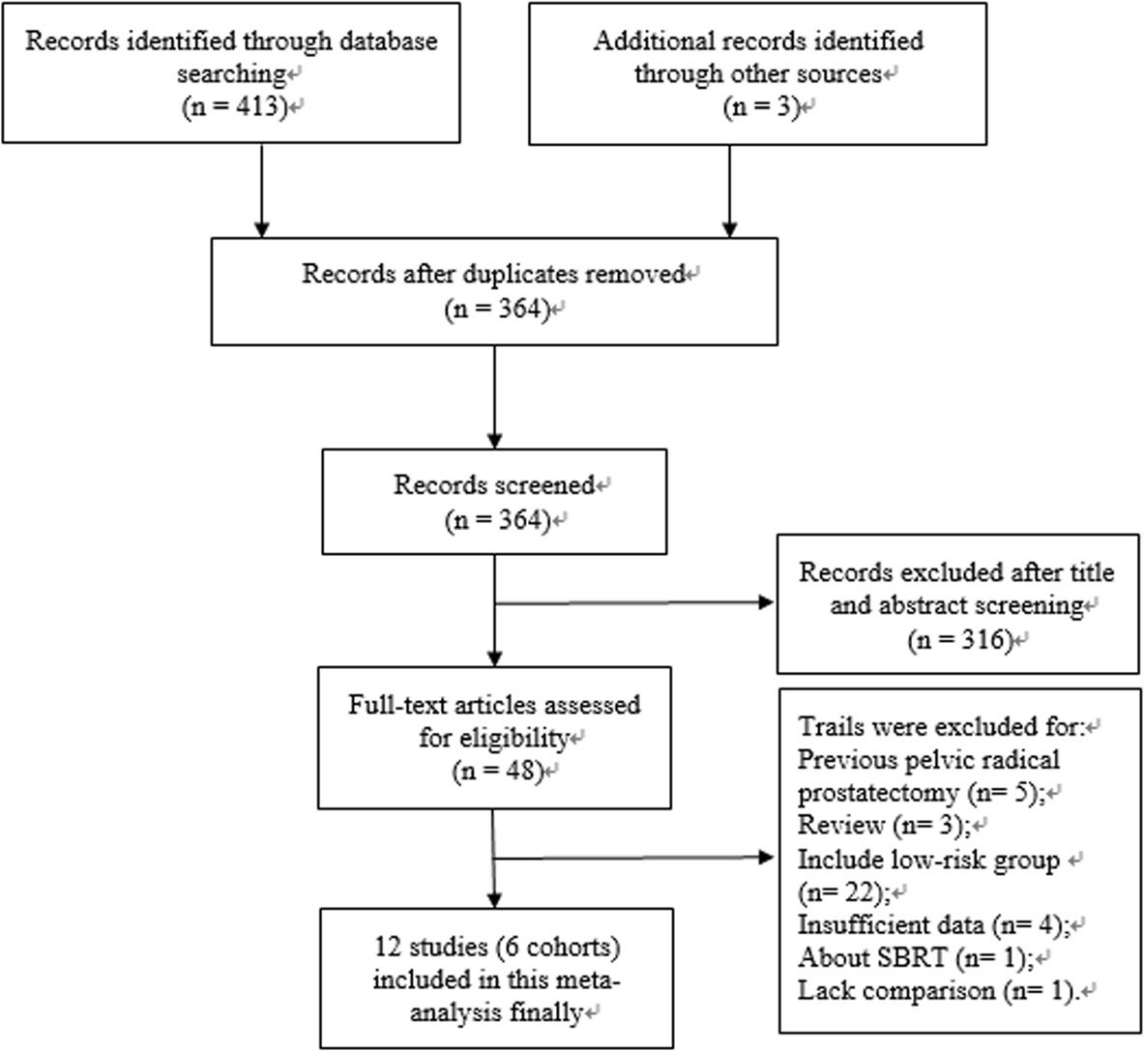
Flow chart of the included trials

A total of 416 articles were initially identified. Duplicates were removed and 364 articles remained. A total of 316 records were excluded after titles and abstracts screening. Full texts and data integrity were then reviewed, and another 36 papers were excluded. In the end, 12 studies (6 cohorts) [12, 13, 16–25] were included in the final meta-analysis. Our article selection process is shown in Fig. 1. All the studies included were randomized controlled trials. Publication years of the records included articles from 2006 to 2017. A total of 2827 patients consisting of 1444 cases treated with hypofractionated radiotherapy and 1383 cases treated with conventional radiotherapy from 6 cohorts were included for this meta-analysis. All patients suffered from intermediate- to high-risk PCa and did not receive previous pelvic radiotherapy or radical prostatectomy. Three cohorts were from Italy, one from the USA and one from Netherlands. The latest study was conducted in 27 centers (14 in Canada, 12 in Australia, and one in France). The detailed characteristics of the selected studies are shown in Table 1.

**Table 1:** Study characteristics

Study	Year	Country	n	TNM or risk group	RT	Design	Schedule	ADT	Outcomes
Aluwini et al	2015–2016	Netherlands	410	T_1b_-T_4_N_X-0_M_X-0_ intermediate- to high-risk	Most IMRT	Hypofractionated versus conventional	64.6Gy (19 fractions within 6.5wks)	Yes	OS, BF acute and late adverse events
			410				78Gy (39 fractions within 8wks)		
Arcangeli et al	2010–2017	Italy	83	≥T_2c_, Gleason ≥7 PSA ≥20ng/ml high-risk	3D-CRT	Hypofractionated versus conventional	62Gy (20 fractions of 3.1Gy, 5wks)	Yes	OS, BF, PCaSS acute and late adverse events
			85				80Gy (40 fractions of 2Gy, 8wks)		
Pollack et al	2007–2013	US	154	T_1_-T_3_, Gleason ≥5 intermediate- to high-risk	IMRT	Hypofractionated versus conventional	70.2Gy (26 fractions of 2.7Gy)	Yes	OS, BF late adverse event
			153				76Gy (38 fractions of 2Gy)		
Marzi et al	2009	Italy	57	≥T_2c_, Gleason7-10 PSA>10ng/ml high-risk	3D-CRT	Hypofractionated versus conventional	62Gy (20 fractions of 3.1Gy)	Yes	late adverse event
			57				80Gy (40 fractions of 2Gy)		
Strigary et al	2009	Italy	80	localized prostate cancer high-risk	3D-CRT	Hypofractionated versus conventional	62Gy (20 fractions of 3.1Gy)	Yes	acute adverse event
			52				56Gy (16 fractions of 3.5Gy)		
							80Gy (40 fractions of 2Gy, 8wks)		
			80						
Catton et al	2017	Canada	608	intermediate-risk	IMRT	Hypofractionated versus conventional	60Gy (20 fractions of 3Gy)	Yes	BF, PCaSS acute and late adverse events
		Australia							
		France	598				78Gy (39 fractions of 2Gy)		

*OS* Overall survival, *BF* Biochemical failure, *ADT* Androgen deprivation therapy, *PCaSS* Prostate cancer-specific survival, *IMRT* Intensity-modulated radiation therapy, *3D-CRT* Three-dimensional conformal radiotherapy, *PSA* Prostate-specific antigen

### Overall survival, prostate cancer-specific survival and biochemical failure

**Fig. 2:**
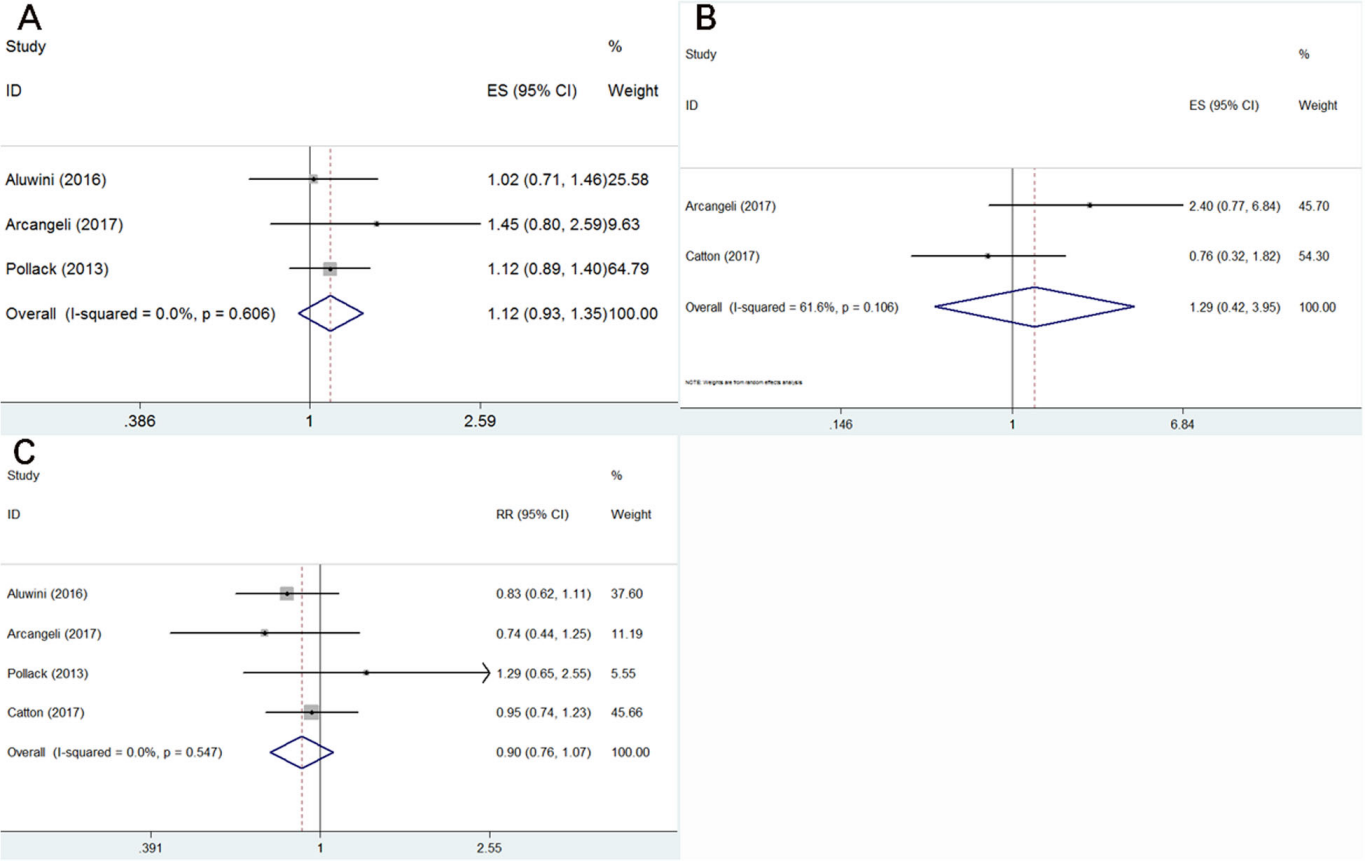
Forest plot for overall survival (**a**), prostate cancer-specific survival (**b**) and biochemical failure (**c**)

Because of homogeneous outcomes of the selected studies (I^2^ = 0, *p* = 0.606), the fixed-effect model was applied for the overall survival (OS) rate. Our results showed that hypofractionated radiotherapy was not superior to conventional radiotherapy (HR = 1.12, 95% CI: 0.93–1.35, *p* = 0.219, Fig. 2a). The hypofractionated radiotherapy and the conventional radiotherapy of patients showed no substantial differences in prostate cancer-specific survival analysis (HR = 1.29, 95% CI: 0.42–3.95, *p* = 0.661) and showed a high level of heterogeneity based on the random effect model (I^2^ = 61.6%, *p* = 0.106, Fig. 2b). We used a fixed-effect model to analyze biochemical failure (BF) because there was no statistical heterogeneity across studies (I^2^ = 0, *p* = 0.440), and the number of patients who were affected by BF was similar among the two groups (RR = 0.90, 95% CI: 0.76–1.07, *p* = 0.248, Fig. 2c).

### Acute and late adverse events

**Fig. 3:**
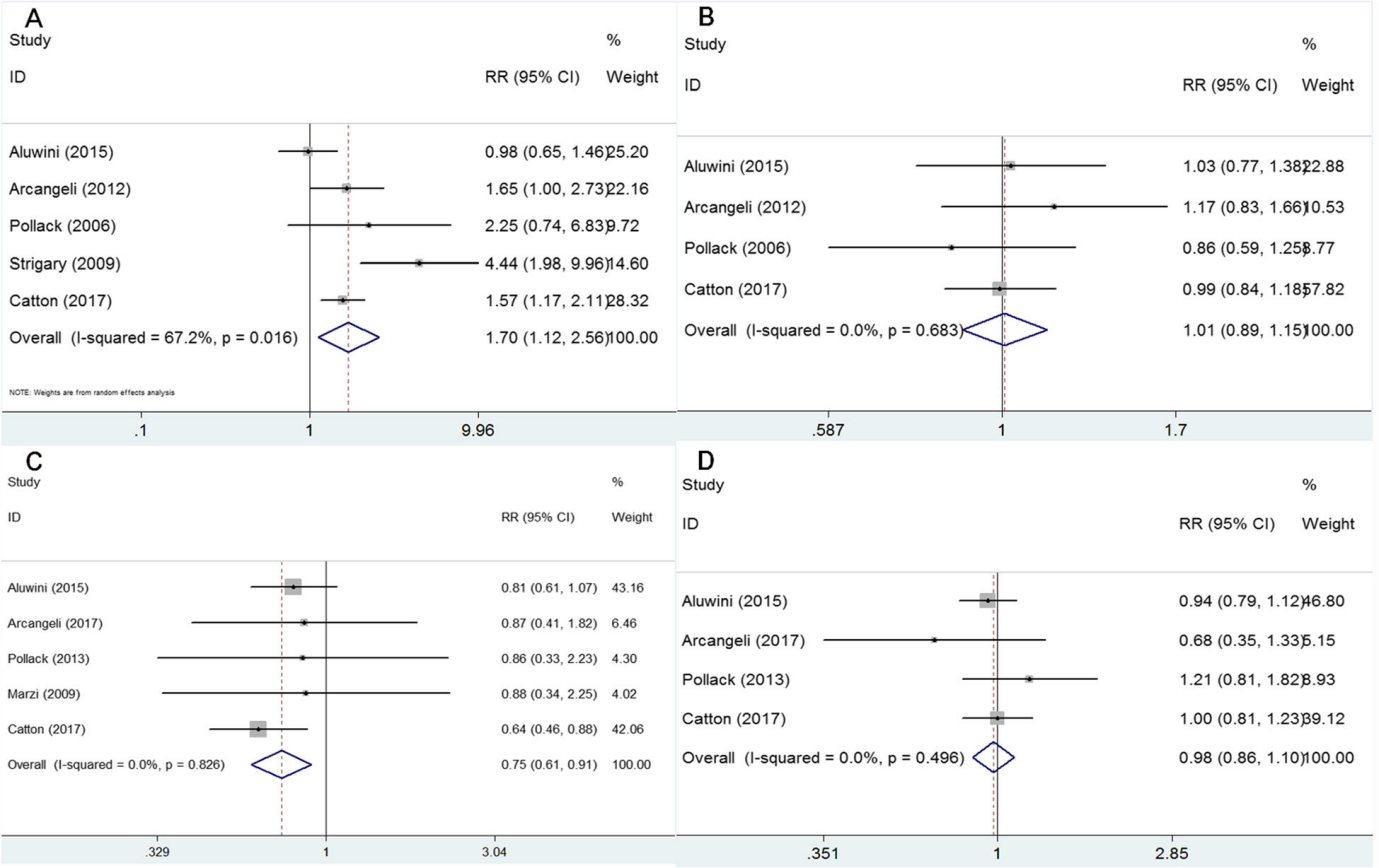
Forest plot for acute adverse gastrointestinal event (**a**), acute genitourinary adverse event (**b**), late adverse gastrointestinal event (**c**) and late adverse genitourinary event (**d**)

The incidence of grade 2 or worse acute adverse gastrointestinal events were analyzed by the random effect model due to heterogeneous outcomes (I^2^ = 67.2%, *p* = 0.016) and the pooled data revealed a clear rising trend in the hypofractionated radiotherapy compared with conventional radiotherapy (RR = 1.70, 95% CI: 1.12–2.56, *p* = 0.012, Fig. 3a). However, acute grade ≥ 3 adverse gastrointestinal events were not significantly different between groups (*p*>0.05). Acute genitourinary adverse events (grade ≥ 2) were similar among the groups (RR = 1.01, 95% CI: 0.89–1.15, *p* = 0.894, Fig. 3b) with no heterogeneity (I^2^ = 0, *p* = 0.683).

Analysis by the fixed-effect model (I^2^ = 0, *p* = 0.826) showed that conventional radiotherapy significantly increased the grade ≥ 2 late gastrointestinal adverse event in comparison with the hypofractionated radiotherapy (RR = 0.75, 95% CI: 0.61–0.91, *p* = 0.003, Fig. 3c). The grade ≥ 2 late genitourinary adverse event data were similar between the hypofractionated radiotherapy and conventional radiotherapy groups (RR = 0.98, 95% CI: 0.86–1.10, *p* = 0.692, Fig. 3d) and no heterogeneity was found for this analysis (I^2^ = 0, *p* = 0.496).

### Subgroup analysis

When we analyzed the subgroup of patients who received only conventional higher doses of radiotherapy (≥78 Gy) versus hypofractionated radiotherapy, the incidence of grade 2 or worse acute adverse gastrointestinal events were still higher in the hypofractionated radiotherapy (RR = 1.66, 95% CI: 1.05–2.61, *p* = 0.029). However, the other results (OS, BF and genitourinary adverse events etc.) were not significantly different between the two groups (all *p*>0.05).

### Sensitivity analysis and publication bias

**Fig. 4:**
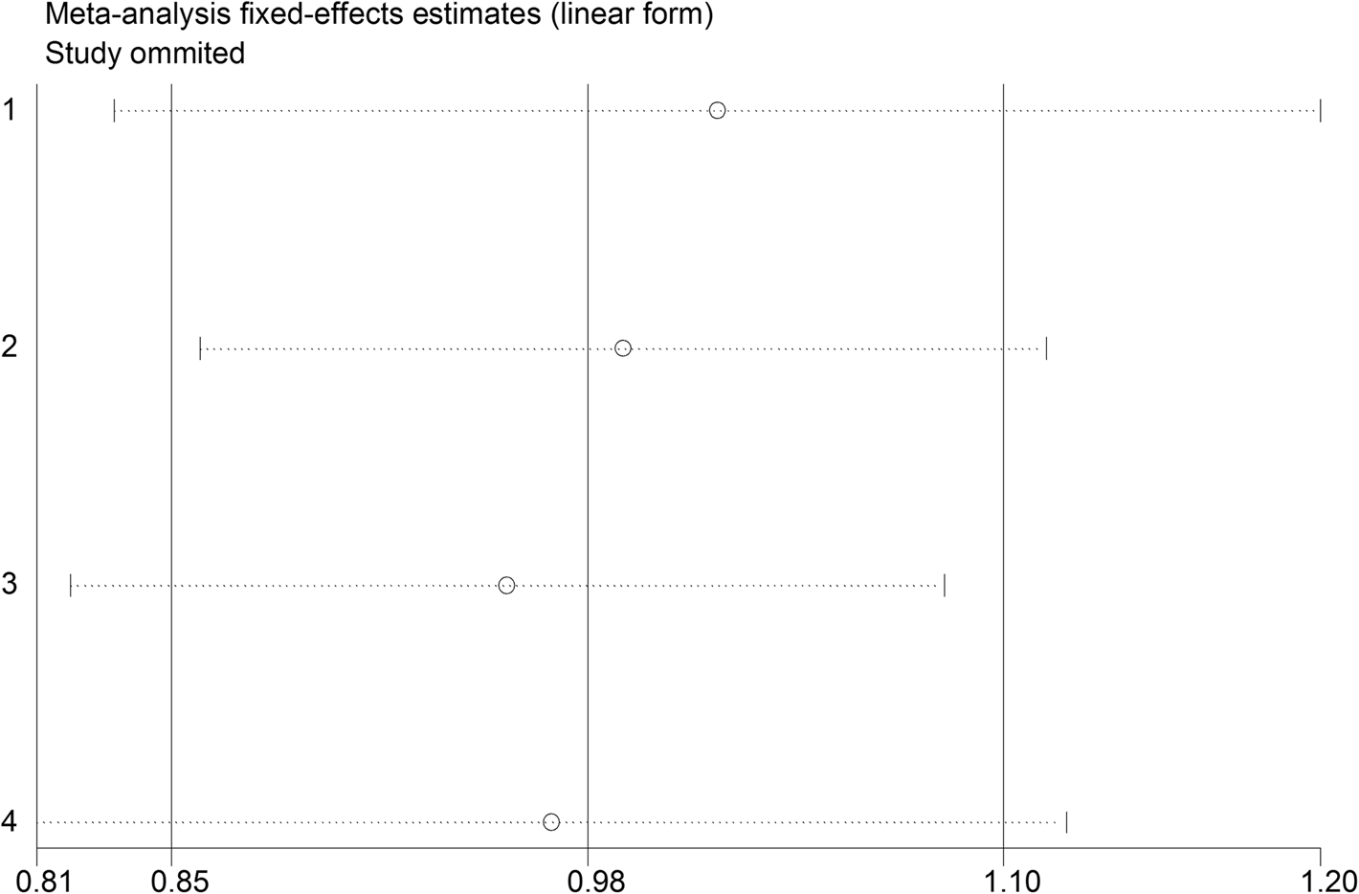
Sensitivity analysis of late adverse genitourinary event

Sensitivity analysis was performed to demonstrate whether the meta-analysis result was robust. The results of sensitivity analysis were shown in Fig. 4, which revealed that no individual studies affected the pooled HR or RR significantly, showing a statistically stability result. Begg test demonstrated no significant statistical evidence of publication bias (*p*>0.05), which suggested that this meta-analysis was not significantly affected by publication bias.

## Discussion

A large number of clinical studies have suggested that dose escalation is associated with improved biochemical and OS outcomes [26–29]. A study of the National Cancer Data Base showed that dose escalation resulted in an improvement in OS for patients with intermediate- to high-risk PCa [30]. Kuban et al. [29] published their dose-escalation study of 301 patients with T_1b_ to T_3_ PCa. Clinical failure or freedom from biochemical was superior for patients treated with 78Gy versus 70Gy (78% vs. 59%, *p* = 0.004), and the patients with initial prostate specific antigen (PSA)>10 ng/ml (intermediate- to high-risk PCa) had a greater benefit (78% vs. 39%, *p* = 0.001). However, conventionally fractionated dose escalation increased toxicity and overall treatment time. With improved radiotherapy technologies, hypofractionated radiotherapy plays a crucial role in the treatment of intermediate- to high-risk PCa. Several randomized trials have proved that efficacy and adverse events of hypofractionated radiotherapy were similar to conventional radiotherapy in most [13, 20] but not all trials [18]. With aims to provide sufficient evidence for clarifying the discrepancies, the present meta-analysis was designed to compare clinical outcomes and adverse events of hypofractionated radiotherapy with conventional radiotherapy for patients with intermediate- to high-risk PCa with the aim to increase the precision of the comparisons and the estimate of treatment benefit.

Overall survival is the most important result for any cancer therapy because it accounts for secondary mortality causes, the interventions used, and all other mortality causes. Given the indolent nature of the progression of prostate cancer, long-term follow-up is of particular importance to assess differences in overall survival [31]. The median follow-up for the selected studies ranges from 5 to 9 years, and we found that hypofractionated radiotherapy was not superior to conventional radiotherapy. Although hypofractionated radiotherapy did not significantly improve overall survival, it enhanced biological efficacy of delivered radiation dose and reduced overall treatment time, presumably making the treatment more acceptable for patients. Biochemical failure was defined according to the Phoenix definition of nadir PSA plus 2 ng/ml [32]. Although there was no significant difference in avoiding biochemical failure between the two groups, there was still a trend in favor of hypofractionated radiotherapy. The α/β ratio for PCa is 1.5Gy from the included studies. After further analysis, we found that the biologically effective dose (BED) of hypofractionated radiotherapy was slightly higher compared to conventional radiotherapy. This difference may explain why no significant difference in biochemical failure was detected between groups.

Recently, hypofractionated radiotherapy has been introduced as treatment for prostate cancer. Noteworthy, hypofractionated radiotherapy schedules have a large variability in the treatment regimens, and the data on adverse events are sparse. Thus, we pooled the relevant data and found the incidence of acute adverse gastrointestinal event (grade ≥ 2) was higher in the hypofractionated radiotherapy; conversely, for late grade ≥ 2 gastrointestinal adverse events, a significant increase in the conventional radiotherapy was found. Furthermore, grade ≥ 3 acute gastrointestinal adverse events in the two groups was not significantly different, and grade 2 acute gastrointestinal adverse events were acceptable for patients. The BED for acute gastrointestinal effect for hypofractionated radiotherapy was significantly greater compared to conventional radiotherapy in the included trials evaluated for acute gastrointestinal toxicity (*p<*0.05). This could be expected to contribute to the increased acute toxicity with hypofractionated radiotherapy. The reduction in late adverse event for hypofractionated radiotherapy is consistent with the linear-quadratic model by Catton et al. [25] that would predict a lower biologically equivalent dose for normal tissues with an α/β of 3-5Gy. This finding is further supported by the trial conducted by Dearnaley et al. [33], who reported a lower 5-year incidence of grade ≥ 2 gastrointestinal adverse events for both hypofractionated groups compared to conventional therapy.

Our pooled data showed that Grade ≥ 2 acute and late genitourinary adverse events were not significantly different between the groups. In 2016, another meta-analysis from Cao et al. found similar genitourinary adverse events between hypofractionated and conventional groups [34]. A long-term late adverse event finding from Arcangeli et al. showed that, a relevant impact did not appear with high-dose fractions and; significant differences were only seen for minor (grade 1), late genitourinary adverse events, namely, for macroscopic hematuria [21].

Our meta-analysis was the first designed to compare clinical outcomes and adverse events between hypofractionated radiotherapy and conventional radiotherapy for the treatment of intermediate- to high-risk localized PCa. In terms of efficacy and adverse events, a large number of studies had tested hypofractionated radiotherapy and found that effects were compared to conventional radiotherapy in the treatment of low-risk localized PCa [35–37]. Published meta-analyses suggest that hypofractionated radiotherapy could result in comparable therapeutic effects for patients suffering from localized prostate cancer without increasing the rate of acute or late adverse events of the gastrointestinal or genitourinary system [38–41]. Our results are in accordance with these previous findings.

Noteworthy, the current meta-analysis had a number of limitations. First, the patients included in our meta-analysis were all Caucasian ethnicity. Therefore, the conclusions of this study should be treated with caution when applied on other ethnic populations. Second, we failed to analyze the absence of biochemical failure because the reported data was insufficient. Third, the heterogeneity of acute gastrointestinal adverse events was relatively large, which might affect its result.

## Conclusion

In summary, meta-analytical data suggest that the efficacy of hypofractionated radiotherapy is comparable to conventional radiotherapy in the treatment of intermediate- to high-risk localized PCa. Although incidences of acute gastrointestinal adverse events were found higher for patients treated with hypofractionated radiotherapy, hypofractionated radiotherapy was safe with overall acceptable adverse event rates. However, due to the limited sample of trials that informed this meta-analysis, these findings should be utilized cautiously when directed in clinical treatment.

## Data Availability

All data are available from the references provided.

## Abbreviations

ADT: Androgen deprivation therapy
BF: Biochemical failure
CI: Confidence interval
EBRT: External-beam radiation therapy
HR: Hazard ratio
OS: Overall survival
PCa: Prostate cancer
PRISMA: Preferred Reporting Items For Systematic Reviews and Meta-analyses
PSA: Prostate specific antigen
RR: Risk ratio

## References

[1] Torre LA, Bray F, Siegel RL, Ferlay J, Lortet-Tieulent J, Jemal A (2015). Global cancer statistics, 2012. CA Cancer J Clin.

[2] Serrano NA, Anscher MS (2016). Favorable vs unfavorable intermediate-risk prostate cancer: a review of the new classification system and its impact on treatment recommendations. Oncology (Williston Park).

[3] Thomsen FB, Mikkelsen MK, Hansen RB (2016). Clinical characteristics and primary management of patients diagnosed with prostate cancer between 2007 and 2013: status from a Danish primary referral center. Acta Oncol.

[4] D'Amico AV, Chen MH, Renshaw A, Loffredo M, Kantoff PW (2015). Long-term follow-up of a randomized trial of radiation with or without androgen deprivation therapy for localized prostate cancer. JAMA.

[5] Pisansky TM, Hunt D, Gomella LG (2015). Duration of androgen suppression before radiotherapy for localized prostate cancer: radiation therapy oncology group randomized clinical trial 9910. J Clin Oncol.

[6] Xu N, Rossi PJ, Jani AB (2011). Toxicity analysis of dose escalation from 75.6 gy to 81.0 gy in prostate cancer. Am J Clin Oncol.

[7] Zelefsky MJ, Levin EJ, Hunt M (2008). Incidence of late rectal and urinary toxicities after three-dimensional conformal radiotherapy and intensity-modulated radiotherapy for localized prostate cancer. Int J Radiat Oncol Biol Phys.

[8] Eade TN, Hanlon AL, Horwitz EM, Buyyounouski MK, Hanks GE, Pollack A (2007). What dose of external-beam radiation is high enough for prostate cancer?. Int J Radiat Oncol Biol Phys.

[9] Miralbell R, Roberts SA, Zubizarreta E, Hendry JH (2012). Dose-fractionation sensitivity of prostate cancer deduced from radiotherapy outcomes of 5,969 patients in seven international institutional datasets: alpha/beta = 1.4 (0.9-2.2) Gy. Int J Radiat Oncol Biol Phys.

[10] Miles EF, Lee WR (2008). Hypofractionation for prostate cancer: a critical review. Semin Radiat Oncol.

[11] Kupelian PA, Reddy CA, Klein EA, Willoughby TR (2001). Short-course intensity-modulated radiotherapy (70 GY at 2.5 GY per fraction) for localized prostate cancer: preliminary results on late toxicity and quality of life. Int J Radiat Oncol Biol Phys.

[12] Arcangeli G, Fowler J, Gomellini S (2011). Acute and late toxicity in a randomized trial of conventional versus hypofractionated three-dimensional conformal radiotherapy for prostate cancer. Int J Radiat Oncol Biol Phys.

[13] Pollack A, Walker G, Horwitz EM (2013). Randomized trial of hypofractionated external-beam radiotherapy for prostate cancer. J Clin Oncol.

[14] Liberati A, Altman DG, Tetzlaff J (2009). The PRISMA statement for reporting systematic reviews and meta-analyses of studies that evaluate health care interventions: explanation and elaboration. Ann Intern Med.

[15] Tierney JF, Stewart LA, Ghersi D, Burdett S, Sydes MR (2007). Practical methods for incorporating summary time-to-event data into meta-analysis. Trials.

[16] Incrocci L, Wortel RC, Alemayehu WG (2016). Hypofractionated versus conventionally fractionated radiotherapy for patients with localised prostate cancer (HYPRO): final efficacy results from a randomised, multicentre, open-label, phase 3 trial. Lancet Oncol.

[17] Aluwini S, Pos F, Schimmel E (2016). Hypofractionated versus conventionally fractionated radiotherapy for patients with prostate cancer (HYPRO): late toxicity results from a randomised, non-inferiority, phase 3 trial. Lancet Oncol.

[18] Aluwini S, Pos F, Schimmel E (2015). Hypofractionated versus conventionally fractionated radiotherapy for patients with prostate cancer (HYPRO): acute toxicity results from a randomised non-inferiority phase 3 trial. Lancet Oncol.

[19] Arcangeli G, Saracino B, Gomellini S (2010). A prospective phase III randomized trial of hypofractionation versus conventional fractionation in patients with high-risk prostate cancer. Int J Radiat Oncol Biol Phys.

[20] Arcangeli S, Strigari L, Gomellini S (2012). Updated results and patterns of failure in a randomized hypofractionation trial for high-risk prostate cancer. Int J Radiat Oncol Biol Phys.

[21] Arcangeli G, Saracino B, Arcangeli S (2017). Moderate hypofractionation in high-risk, organ-confined prostate cancer: final results of a phase III randomized trial. J Clin Oncol.

[22] Pollack A, Hanlon AL, Horwitz EM (2006). Dosimetry and preliminary acute toxicity in the first 100 men treated for prostate cancer on a randomized hypofractionation dose escalation trial. Int J Radiat Oncol Biol Phys.

[23] Marzi S, Saracino B, Petrongari MG (2009). Modeling of alpha/beta for late rectal toxicity from a randomized phase II study: conventional versus hypofractionated scheme for localized prostate cancer. J Exp Clin Cancer Res.

[24] Strigari L, Arcangeli G, Arcangeli S, Benassi M (2009). Mathematical model for evaluating incidence of acute rectal toxicity during conventional or hypofractionated radiotherapy courses for prostate cancer. Int J Radiat Oncol Biol Phys.

[25] Catton CN, Lukka H, Gu CS (2017). Randomized trial of a Hypofractionated radiation regimen for the treatment of localized prostate cancer. J Clin Oncol.

[26] Peeters ST, Heemsbergen WD, Koper PC (2006). Dose-response in radiotherapy for localized prostate cancer: results of the Dutch multicenter randomized phase III trial comparing 68 Gy of radiotherapy with 78 Gy. J Clin Oncol.

[27] Dearnaley DP, Jovic G, Syndikus I (2014). Escalated-dose versus control-dose conformal radiotherapy for prostate cancer: long-term results from the MRC RT01 randomised controlled trial. Lancet Oncol.

[28] Denham JW, Steigler A, Joseph D (2015). Radiation dose escalation or longer androgen suppression for locally advanced prostate cancer? Data from the TROG 03.04 RADAR trial. Radiother Oncol.

[29] Kuban DA, Tucker SL, Dong L (2008). Long-term results of the M. D. Anderson randomized dose-escalation trial for prostate cancer. Int J Radiat Oncol Biol Phys.

[30] Kalbasi A, Li J, Berman A (2015). Dose-escalated irradiation and overall survival in men with nonmetastatic prostate cancer. JAMA Oncol.

[31] Morgan SC, Waldron TS, Eapen L (2008). Adjuvant radiotherapy following radical prostatectomy for pathologic T3 or margin-positive prostate cancer: a systematic review and meta-analysis. Radiother Oncol.

[32] Roach M, Hanks G, Thames H (2006). Defining biochemical failure following radiotherapy with or without hormonal therapy in men with clinically localized prostate cancer: recommendations of the RTOG-ASTRO Phoenix consensus conference. Int J Radiat Oncol Biol Phys.

[33] Dearnaley D, Syndikus I, Mossop H (2016). Conventional versus hypofractionated high-dose intensity-modulated radiotherapy for prostate cancer: 5-year outcomes of the randomised, non-inferiority, phase 3 CHHiP trial. Lancet Oncol.

[34] Cao L, Yang YJ, Li ZW (2017). Moderate hypofractionated radiotherapy is more effective and safe for localized prostate cancer patients: a meta-analysis. Oncotarget.

[35] Valeriani M (2014). Image-guided hypofractionated radiotherapy in low-risk prostate cancer patients. J Biomed Biotechnol.

[36] Valeriani M (2018). Moderate hypofractionation in patients with low-risk prostate cancer: long-term outcomes. Anticancer Res.

[37] Bruner DW, et al. Quality of life in patients with low-risk prostate cancer treated with hypofractionated vs conventional radiotherapy: a phase 3 randomized clinical trial. JAMA Oncol, Epub ahead of print. 2019. 10.1001/jamaoncol.2018.6752 PMC6459051 30763425

[38] Yin ZZ, You JQ, Wang YY (2019). Moderate hypofractionated radiotherapy vs conventional fractionated radiotherapy in localized prostate cancer: a systemic review and meta-analysis from phase III randomized trials. Onco Targets Ther.

[39] Carvalho ÍT, Baccaglini W, Claros OR (2018). Genitourinary and gastrointestinal toxicity among patients with localized prostate cancer treated with conventional versus moderately hypofractionated radiation therapy: systematic review and meta-analysis. Acta Oncol.

[40] Sánchez-Gómez LM, Polo-deSantos M, Rodríguez-Melcón JI (2015). Hypofractionated radiation therapy versus conventional radiation therapy in prostate cancer: a systematic review of its safety and efficacy. Actas Urol Esp.

[41] Botrel TE, Clark O, Pompeo AC (2013). Hypofractionated external-beam radiation therapy (HEBRT) versus conventional external-beam radiation (CEBRT) in patients with localized prostate cancer: a systematic review and meta-analysis. Core Evid.

